# Unanticipated functional diversity among the TatA-type components of the Tat protein translocase

**DOI:** 10.1038/s41598-018-19640-3

**Published:** 2018-01-22

**Authors:** Ekaterina Eimer, Wei-Chun Kao, Julia Fröbel, Anne-Sophie Blümmel, Carola Hunte, Matthias Müller

**Affiliations:** 1 Institute of Biochemistry and Molecular Biology, ZBMZ, Faculty of Medicine, University of Freiburg, 79104 Freiburg, Germany; 2 Faculty of Biology, University of Freiburg, 79104 Freiburg, Germany; 3 Spemann Graduate School of Biology and Medicine (SGBM), University of Freiburg, 79104 Freiburg, Germany; 4 BIOSS Centre for Biological Signalling Studies, University of Freiburg, 79104 Freiburg, Germany

## Abstract

Twin-arginine translocation (Tat) systems transport folded proteins that harbor a conserved arginine pair in their signal peptides. They assemble from hexahelical TatC-type and single-spanning TatA-type proteins. Many Tat systems comprise two functionally diverse, TatA-type proteins, denominated TatA and TatB. Some bacteria in addition express TatE, which thus far has been characterized as a functional surrogate of TatA. For the Tat system of *Escherichia coli* we demonstrate here that different from TatA but rather like TatB, TatE contacts a Tat signal peptide independently of the proton-motive force and restricts the premature processing of a Tat signal peptide. Furthermore, TatE embarks at the transmembrane helix five of TatC where it becomes so closely spaced to TatB that both proteins can be covalently linked by a zero-space cross-linker. Our results suggest that in addition to TatB and TatC, TatE is a further component of the Tat substrate receptor complex. Consistent with TatE being an autonomous TatAB-type protein, a bioinformatics analysis revealed a relatively broad distribution of the *tatE* gene in bacterial phyla and highlighted unique protein sequence features of TatE orthologs.

## Introduction

Bacteria, archaea, and plant chloroplasts have the capability to transport precursor proteins in a folded state across membranes. Precursor proteins that qualify for this mode of transport are primarily distinguished by an SRRxFLK sequence motif in the N-terminal part of their signal sequences. This consensus motif, of which the double arginine is largely invariant, is recognized by so-called Tat translocases (Tat stands for twin-arginine translocation). A second determinant for the specific recognition of signal peptides by Tat translocases is the hydrophobicity of their core region^1,2^.

Tat translocases do not pre-exist in the membrane as stable protein complexes but rather assemble on demand^3,4^ from TatA-type and TatC-type membrane proteins. The TatC subunits of Tat translocases possess six transmembrane helices^5,6^. They associate with a varying number of homologous TatA-type proteins, whose N-terminal structure is characterized by a single transmembrane helix followed by an amphipathic domain^7–10^. Our model organism, the Gram-negative bacterium *Escherichia coli*, expresses three TatA-type proteins, TatA, TatB and TatE. As per degree of homology, TatA and TatB evolved early from a common ancestor whilst TatE emerged from TatA by a late gene duplication event.

TatC functions as the primary docking site for the Tat signal peptide at the Tat translocase^11,12^ directly recognizing the RR-consensus motif^13^. TatC provides binding sites for TatA and TatB^12,14–16^. In concert with TatB, TatC forms a dome-shaped intramembrane binding cavity allowing the hairpin-like insertion of the Tat signal peptide^14,17^.

Despite their structural homology, TatA and TatB have distinct, non- interchangeable functions during Tat-dependent translocation. By interacting with two different sites on TatC, TatB links neighboring TatC monomers thereby forming circular, hetero-multimeric substrate receptor complexes^14,15^. TatB interacts with the Tat signal peptide downstream of the RR-consensus motif in a proton-motive force independent manner^11,14,18^. In addition, it functions as a major binding platform for the folded mature domain of Tat substrates^19^.

TatA is more abundant than all the other Tat subunits with the actual stoichiometry, however, being at issue^20–22^. Depending on the proton-motive force, TatA associates with the TatBC complex^23^ and the signal peptide^14,24^. It facilitates through its homo-oligomerization^25,26^ the transmembrane translocation of the substrate in a still elusive manner. TatA competes with TatB for binding to TatC^14–16^.

The function of the smallest TatA-type protein of *E. coli*, TatE, remains to be elucidated. *In vivo* studies demonstrated that TatE can maintain Tat transport in the absence of a functional TatA^3,27,28^ suggesting TatE be a functional paralog of TatA. This, however, would be insufficient to explain why TatE has persisted during evolution as an individual isoform. Previously we demonstrated that TatE is a regular constituent of the Tat translocase in *E. coli*^28^. We found TatE to interact with TatA, TatB, and TatC and to localize to active Tat translocases *in vivo*. Here we show that TatE exhibits distinct properties rendering it a functional hybrid between TatA and TatB. Using a bioinformatic approach we demonstrate that *tatE* genes are more abundant among the bacterial kingdom than anticipated, further emphasizing an individual relevance of this Tat subunit.

## Results and Discussion

### TatE and TatB of *E. coli* share functional properties

**Figure 1:**
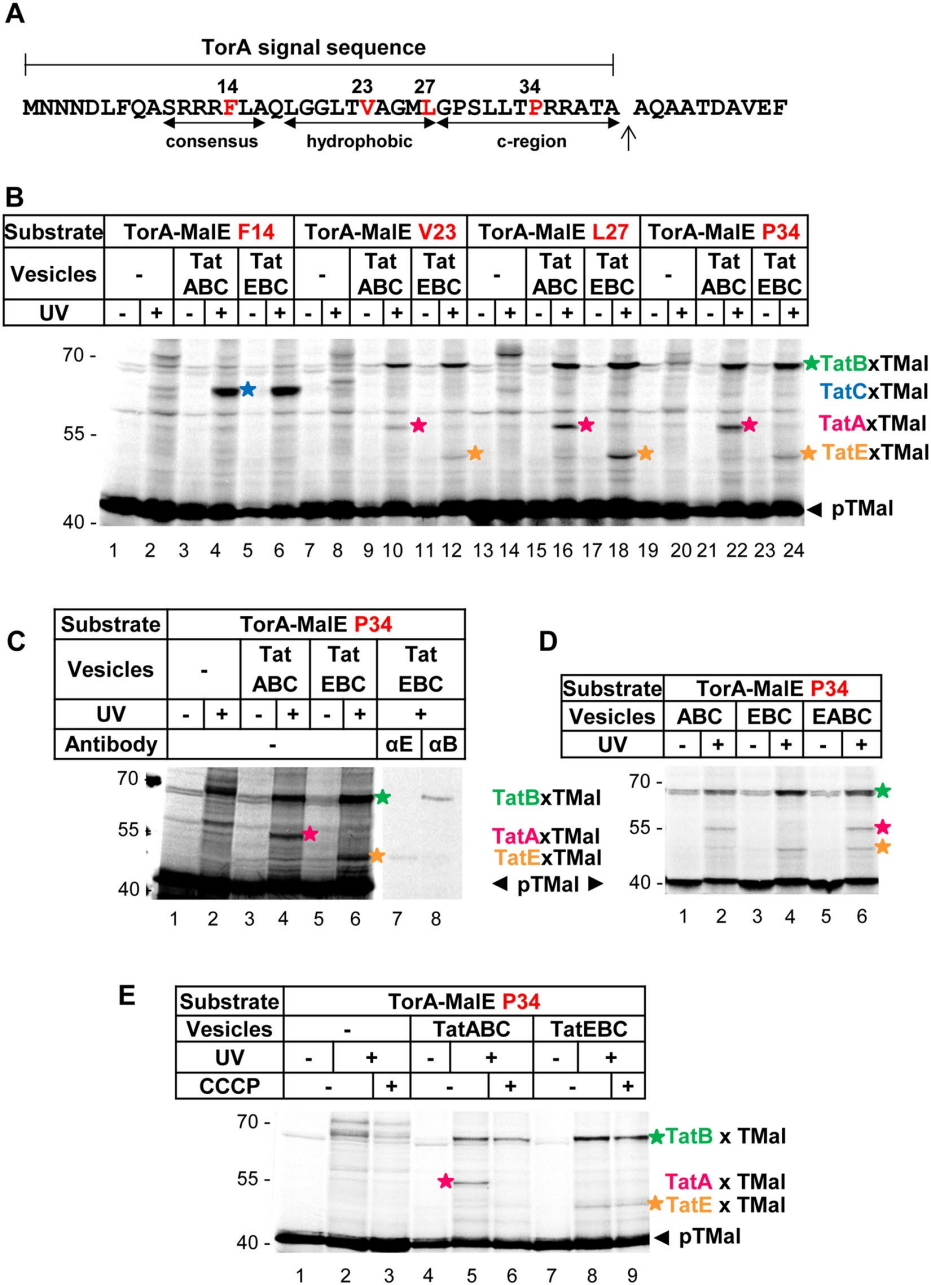
TatE interacts with the signal sequence of a Tat substrate. (**A**) Primary structure of the TorA-signal sequence. The twin-arginine consensus motif, the hydrophobic core and the c-region are indicated. Amino acid residues substituted by the photo-crosslinker *p*-benzoyl-L-phenylalanine (Bpa) are labeled in red. (**B**–**E**) The model Tat substrate TorA-MalE335 (TMal) was *in vitro* synthesized and radioactively labeled. Bpa was incorporated into the signal sequence at the indicated positions (red). After incubation with inverted inner membrane vesicles containing the indicated Tat components, crosslinking was induced by UV-irradiation (UV). The samples were resolved by 10% SDS-PAGE and analyzed by phosphorimaging. Adducts between the substrate and TatC (blue star), TatB (green star), TatA (magenta stars) and TatE (orange stars) are indicated. (**C**) Adducts between TMal, TatE and TatB were confirmed by immuno-precipitation using antisera against TatE and TatB. The eight lanes are derived from a single gel after excising a piece between lanes 6 and 7. (**D**) In vesicles containing TatE alongside TatA, the signal peptide cross-links to both Tat proteins. (**E**) Dissipation of the proton-motive force by 0.1 mM CCCP abolishes contacts with TatA but not with TatE.

We previously demonstrated that TatE displays the properties of a constitutive component of the *E. coli* Tat translocase, as it localizes to functional Tat translocases in living cells and interacts individually with TatA, TatB, and TatC^28^. However, whereas TatA^11,24,29^, TatB^14,18,30^, and TatC^11,13,18^ have all been shown to come into contact with the RR-signal sequence of Tat substrate proteins, direct interactions of TatE with substrate have not yet been demonstrated. We therefore equipped the model Tat substrate TorA-MalE335 with the photo cross-linker *p*-benzoyl-phenylalanine (Bpa). In TorA-MalE335, the RR-signal peptide of the natural *E. coli* Tat substrate TorA (trimethylamine oxide reductase) is fused to a C-terminally truncated version of the periplasmic maltose-binding protein MalE^12,17^. Figure 1A highlights the four positions within the TorA signal peptide that were individually replaced by Bpa during cell-free transcription/translation, using an amber stop codon-based approach. The *in vitro* synthesized Bpa variants of TorA-MalE335 (pTMal) were incubated with inside-out inner membrane vesicles, which had been prepared from a derivative of *E. coli* strain DADE overexpressing various combinations of the plasmid-encoded TatABCE proteins in a *ΔtatABCE* background. When cross-linking was initiated by irradiation with UV-light, the previously described^11,14,18,31^ site-specific interactions with TatC and TatB were obtained. Thus, Bpa located at position F14 within the RR-consensus motif of the TorA signal peptide cross-links to TatC (Fig. 1B, lane 4, blue star), whereas Bpa incorporated into the hydrophobic core and the c-region of the TorA signal sequence (cf. Fig. 1A) yields adducts to TatB and TatA (Fig. 1B, lanes 10, 16, 22, green and red stars, respectively). When in this experimental setup membrane vesicles containing TatEBC instead of TatABC were employed (lanes 6, 12, 18, 24), cross-linking of the TorA signal sequence to TatB and TatC persisted (blue and green stars), whereas the TatA cross-links (red stars) were no longer obtained. Instead, lower molecular mass adducts (orange stars) appeared that by size correspond to a cross-link between TorA-MalE335 and TatE. This was confirmed by immuno-precipitation using antibodies directed towards TatE (Fig. 1C, lane 7). When vesicles contained both TatE and TatA, the signal sequence of TorA-MalE335 was found cross-linked to both Tat subunits (Fig. 1D, lane 6). These findings indicate that in the assembled Tat translocase, TatE locates close to the hydrophobic core and c-region of an RR-signal peptide exactly like TatA and TatB do. No competition between TatE and TatA for interacting with the Tat substrate was observed under these experimental conditions.

A characteristic feature of the cross-links between RR-signal peptides and TatA is their sensitivity towards dissipation of the H^+^-motive force^11^. This is illustrated in Fig. 1E, where the UV-dependent adduct between the TorA-MalE335 precursor and TatA (red star) disappears upon addition of the protonophore cyanide *m*-chlorophenyl-hydrazone (CCCP), whereas the TatB adducts (green stars) persist in the presence of CCCP (lanes 5 and 6). In contrast to TatA and exactly like TatB, TatE was found cross-linked to TorA-MalE335 regardless of whether CCCP was present or not (lanes 8 and 9, orange stars). The results presented in Fig. 1 therefore reveal a property of TatE that would not be expected, if TatE were a functional homologue solely of TatA. Interaction with a Tat signal sequence independently of the H^+^-motive force rather is a typical feature of TatB.

**Figure 2:**
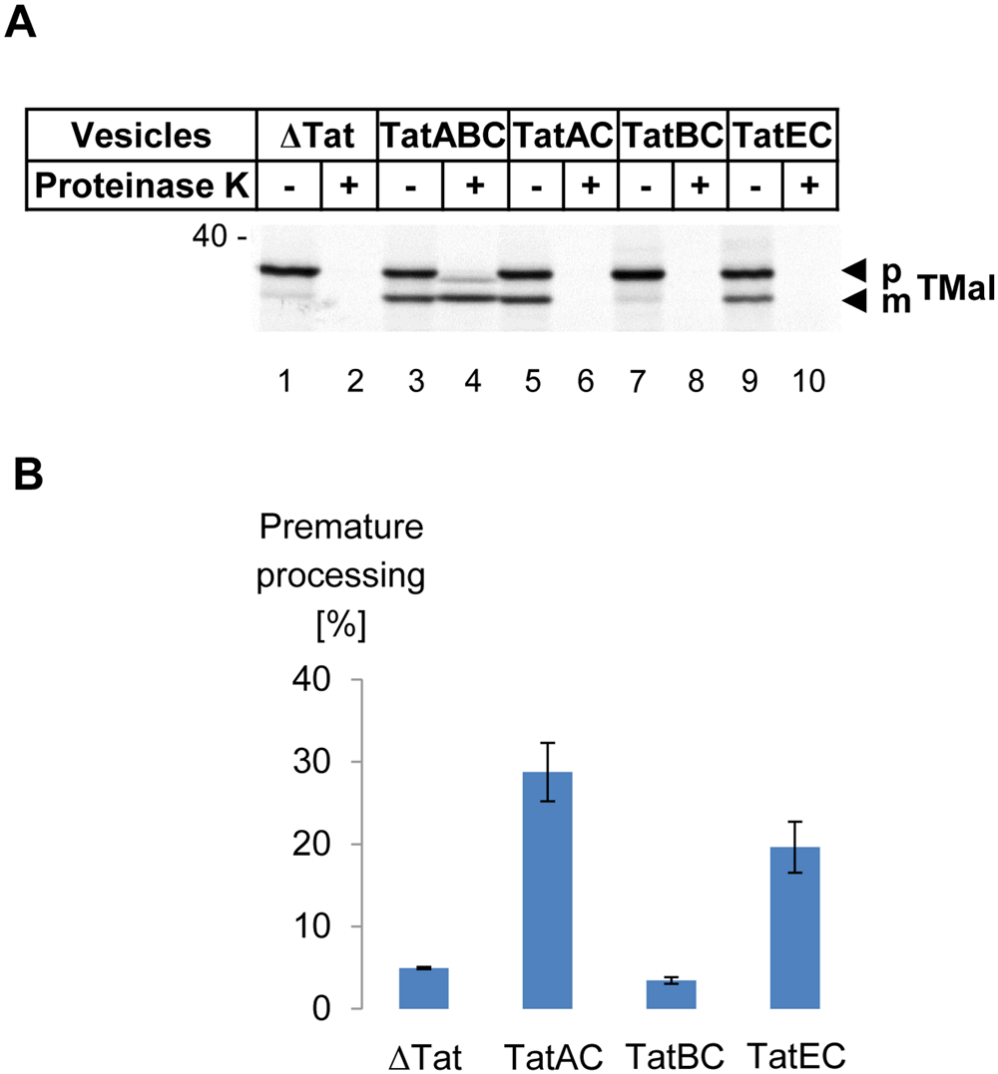
TatE partly prevents premature cleavage of the TorA signal peptide. (**A**) The Tat substrate TorA-MalE335 (TMal) was synthesized and radioactively labeled *in vitro* and incubated with inverted inner membrane vesicles obtained from *E. coli* strain BL21(DE3)*Δtat* expressing the plasmid-encoded Tat components indicated. The samples were resolved by 10% SDS-PAGE and analyzed by phosphorimaging. In the presence of TatABC-containing vesicles, cleavage of the precursor (pTMal) to the mature protein (mTMal) is accompanied by the acquirement of proteinase K resistance (compare lanes 2 and 4) indicating transport into the lumen of the vesicles. Vesicles lacking TatB cleave pTMal (lane 5) but do not allow transport to occur (lane 6, no protease resistance). TatB (lane 7) and to a lesser extent TatE (lane 9) prevent this premature cleavage. (**B**) Quantification of premature processing of pTMal to mTMal by the indicated membrane vesicles was obtained from nine independent experiments using vesicles from two different preparations each, except for ΔTat vesicles (three experiments, one preparation).

We previously reported that in the absence of TatA and TatB, TatC by itself enables RR-signal sequences of Tat substrates to insert into the cytoplasmic membrane of *E. coli*. Insertion was shown to proceed to the point that RR-signal sequences are recognized by signal peptidase and prematurely cleaved without the actual Tat substrates being translocated^17^. In this scenario, a typical feature of TatB, which is not shared by TatA, is to prevent this TatC-mediated premature cleavage of the signal peptide. This is addressed in Fig. 2A. When the precursor form of TorA-MalE335 (pTMal) is synthesized *in vitro* in the presence of membrane vesicles lacking all Tat components (*Δ*Tat), it is completely sensitive towards digestion by proteinase K (compare lanes 1 and 2). In the presence of TatABC-containing vesicles, however, pTMal becomes processed by the signal peptidase of the vesicles to the mature form (lane 3, mTMal), which due to transport into the vesicle lumen is now resistant towards proteinase K (lane 4). Only a minor fraction of uncleaved precursor (pTMal) is translocated under these conditions as indicated by protease resistance (lane 4). Note that the slightly smaller size of the protease-treated pTMal (compare lanes 3 and 4) is the result of proteinase K removing of a few N-terminal amino acids from the membrane-embedded signal peptide of translocated yet non-processed pTMal^17^.

If in these experimental conditions membrane vesicles are used that contain only TatA and TatC (lane 5), roughly 30% of pTMal are still converted to mTMal (Fig. 2B) similar to what is seen with TatABC vesicles (Fig. 2A, compare lanes 3 and 5). As previously shown, the cleavage of pTMal by TatAC vesicles requires an intact RR-motif, i.e. recognition by TatC, as well as a functional signal peptidase cleavage site^17^. Although caused by signal peptidase, cleavage of pTMal is premature, because the TatAC vesicles do not allow translocation, as demonstrated by the accessibility of the cleaved mTMal to proteinase K (lane 6). In contrast, TatBC vesicles lacking TatA do not allow for the conversion of pTMal to mTMal (Fig. 2A, lane 7; Fig. 2B). A minimal cleavage of pTMal by TatBC vesicles to a product slightly bigger in size than mTMal (lane 7) was also observed by vesicles entirely lacking the Tat translocase (lane 1) and is therefore caused by an unknown protease. Prevention of premature processing by TatBC vesicles is consistent with TatB and TatC concertedly forming an intramembrane binding cavity for the RR-signal peptide. Because TatA is not a primary constituent of this binding cavity, it is not able to prevent the signal peptide from crossing the membrane and being prematurely processed by signal peptidase^14,17^. Membrane vesicles harboring only TatE and TatC, however, showed a significantly reduced premature processing of pTMal, although they were not as inhibitory as TatBC vesicles (Fig. 2A, lane 9; Fig. 2B). Thus like TatB and different from TatA, TatE is also able to counteract the TatC-mediated premature cleavage of the Tat substrate, yet with less efficiency than TatB. The data presented in Figs. 1 and 2 collectively suggest that TatE might play a role as part of the TatBC receptor complex for Tat substrates.

### TatE paralogs show distinct sequence motifs and occur also outside of Enterobacteria

**Figure 3:**
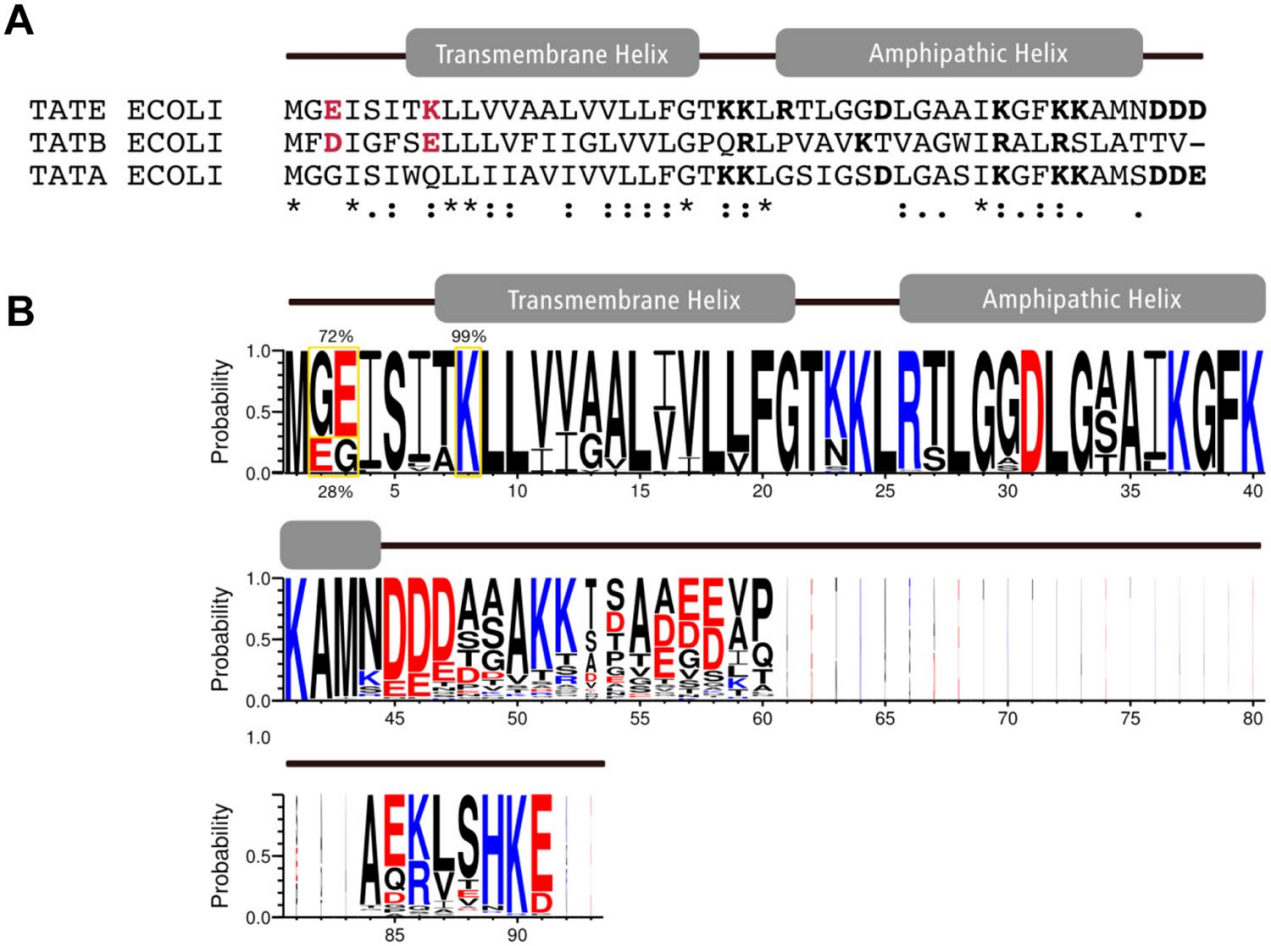
Similar to TatB, TatE has conserved N-terminal charges. (**A**) Sequence alignment of the amino acid sequences of *E. coli* TatE (accession number: P0A843), TatB (P69425) and TatA (P69428). N-terminal, charged amino acid residues of TatE and TatB are highlighted in red, other charged residues are in bold. Secondary structural elements are assigned according to the NMR structure of TatAd from *B. subtilis*^9^ based on the multiple sequence alignment to its *E. coli* homologues (see Fig. 5B). (**B**) Consensus sequence of TatE obtained from the alignment of 111 distinct TatE protein sequences encoded by members of the Family of *Enterobacterales*. The N-terminal charged amino acids are boxed and their degree of conservation is indicated.

A TatB-like function of TatE would also be consistent with the about 50-fold lower expression level of *tatE* compared to *tatA*^20^. Nevertheless, TatE of *E. coli* shows a much higher sequence identity with TatA than with TatB and was shown to partially compensate the phenotype of a *tatA* deletion mutant under certain experimental conditions^3,15,27,28^. Given such a seeming bifunctionality and the fact that TatE has been characterized as a constitutive member of the *E. coli* Tat translocase^28^, TatE of *E. coli* seems to be a distinct member of the TatA family rather than a dormant surrogate for TatA or TatB. Consistent with this assumption, the N-terminal amino acid sequences of TatA, TatB, and TatE from *E. coli* reveal individual differences as manifested by a disparate distribution of charged amino acids (Fig. 3A). TatE and TatB each possess two charged amino acid residues in position 3 (N-tail) and position 8 (transmembrane helix), although the latter one is of opposite polarity (Lys in TatE, Glu, however, in TatB). In contrast, TatA of *E. coli* is uncharged in its N-terminus.

In order to investigate the significance of the two N-terminal charged residues of *E. coli* TatE, we searched the NCBI Reference Sequence protein database for homologous sequences. 3120 sequences were obtained, of which 121 were annotated as TatE, 99 as SecE, 2041 as TatA, and 859 were annotated differently. The sequences annotated as SecE clearly share characteristics with TatE orthologues, whereas SecE is a structurally totally diverse subunit of the functionally unrelated Sec translocase^32^. It is therefore likely that these 99 TatE homologues have erroneously been annotated as SecE in the data base used. For reasons of unambiguity, we, however, excluded them from our analysis.

Among the 121 TatE sequences, 111 are from the Order of *Enterobacterales* that contains the Family of *Enterobacteriaceae*, for which almost exclusively TatE paralogs had hitherto been described. The 10 non-enterobacterial TatE were recovered from the Deltaproteobacterium *Bacteriovorax sp. DB6* and the Gammaproteobacteria *Halotalea alkalilenta* and *Gilliamella apicola*. A consensus sequence model of the complete 111 enterobacterial TatE sequences is shown in Fig. 3B. Lysine at position 8 was found to be conserved to 99%. The majority (72%) of those TatE sequences display glutamate at position 3 with a preceding Gly. In the remaining 28% of sequences, Glu is found at position 2 and Gly at position 3 resulting in either a Gly-Glu or a Glu-Gly pair at this place. Thus in all enterobacterial TatE sequences, the N-termini are distinguished by an E^3^xxxxK^8^ or an E^2^xxxxxK^8^ motif.

In line with similar functional properties of TatE and TatB, an N-proximal negatively charged residue, as found here to be conserved among the enterobacterial TatE orthologs, was previously shown to be associated with TatB-like functions. The Freudl group isolated mutants in *E. coli tatA* that phenotypically suppressed the deletion of TatB. Most of the suppressors had gained an aspartate at the otherwise uncharged N-terminus of TatA and thereby the ability to functionally replace TatB^33^.

**Figure 4:**
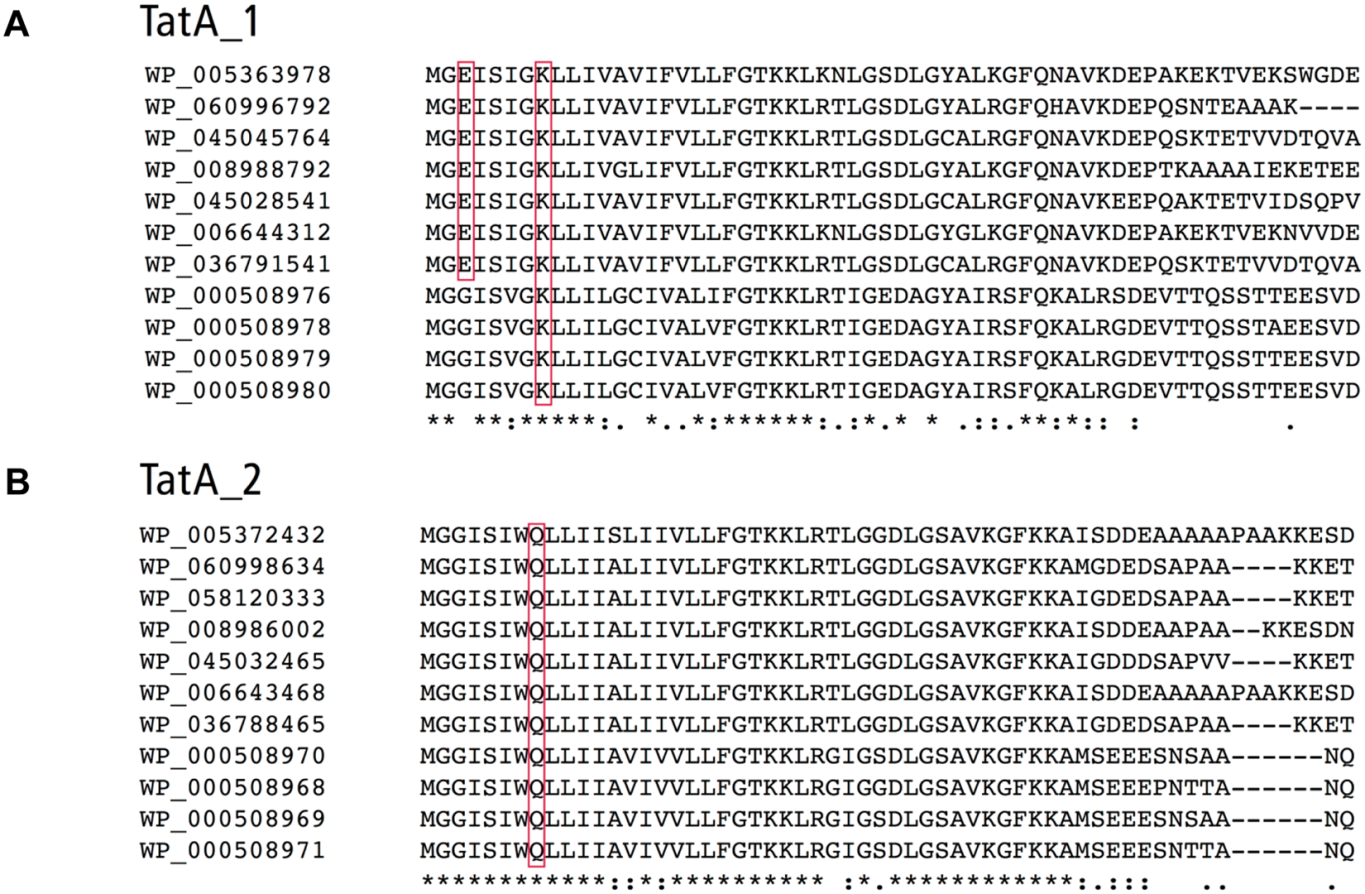
Two distinct TatA orthologs of *Vibrionales*. TatA_1 paralogs (**A**) feature N-terminal charged residues, which are similar to *E. coli* TatE (Glu^3^ and Lys^8^, boxed), or a single lysine at the 8^th^ position. TatA_2 paralogs (**B**) carry no charged residues at the corresponding section. The sole polar residue of TatA_2 is Gln^8^, which is conserved in TatA of *E. coli*. Listed are selected strains of the Order of *Vibrionales* with the following accession numbers of their TatA_1 and TatA _2 paralogs, respectively: *Photobacterium angustum* (WP_005363978, WP_005372432); *Photobacterium aquimaris* (WP_060996792, WP_060998634); *Photobacterium kishitanii* (WP_045045764, WP_058120333); *Photobacterium leiognathi* (WP_008988792, WP_008986002); *Photobacterium phosphoreum* (WP_045028541, WP_045032465); *Photobacterium* sp. SKA34 (WP_006644312, WP_006643468); *Photobacterium* (WP_036791541, WP_036788465); *Vibrio cholerae* (WP_000508976, WP_000508970/WP_000508978, WP_000508968/WP_000508979, WP_000508969/WP_000508980, WP_000508971).

Out of the 3120 sequences obtained from our database search for homologues of the *E. coli* TatE, 2041 entries were annotated as TatA (Table 1), which reflects the high sequence identity between both Tat proteins^15^. These TatE homologues are almost entirely of proteobacterial origin (Table 1). When screened for the occurrence of charged N-terminal amino acids, 108 of those 2041 TatA proteins were found to harbor the E^3^xxxxK^8^ or E^2^xxxxxK^8^ motif, 44 merely a Lys at position 8, whereas 1889 do not carry similar charge patterns in their N-termini. Interestingly, of those bacterial species encoding TatA homologues with the aforementioned charge patterns, 50 possess an additional TatA paralog, of which the N-terminus is not charged. This is exemplified in Fig. 4 for selected species from the Order of *Vibrionales*, of which both types of TatA paralogs were separately aligned. The one with the higher homology to *E. coli* TatE and the shorter length (63–78 amino acids) is labeled TatA_1. Seven of them in fact display the E^3^xxxxK^8^ motif (boxed), whilst all of them possess the Lys at position 8. The second group of TatA paralogs (denominated TatA_2) lacks any N-terminal charges and consistently contains the Gln^8^ of *E. coli* TatA. These findings demonstrate that the occurrence of TatE-type orthologs harboring distinct N-terminal charged amino acids is not limited to enterobacteria but also encompasses other Gamma-proteobacteria. Furthermore, their co-existence in the genomes with TatA-type paralogs, which do not carry TatE-specific N-terminal charge patterns, would support the idea that TatE-type proteins when co-expressed might serve a unique functional purpose.

**Table 1:** Listed are the 2041 TatE homologues annotated as TatA.

Class	Order	Total Hits	N-Charged
Alphaproteobacteria	Rhizobiales	158	
	Sphingomonadales	25	
	Rhodobacterales	14	
	others	2	
Betaproteobacteria	Burkholderiales	339	
	Neisseriales	38	
	Rhodocyclales	26	
	Methylophilales	17	
	others	26	
Gammaproteobacteia	Enterobacterales	443	96
	Pseudomonadales	269	8
	Alteromonadales	153	
	Vibrionales	138	42
	Oceanospirillales	107	1
	Pasteurellales	41	
	Legionellales	39	
	Thiotrichales	27	
	Cellvibrionales	24	
	Xanthomonadales	22	
	Aeromonadales	21	2
	Chromatiales	19	
	Methylococcales	16	3
	others	33	
Epsilonproteobacteria	Campylobacterales	9	
	Sulfurovum	4	
Deltaproteobacteria		5	
All others		26	
		2041	152

The sequences are sorted by the Class of the source strains, the number of sequences found in each Order, and the occurrence of N-terminal charges (including 108 entries with E^3^xxxxK^8^ or E^2^xxxxxK^8^ motifs and 44 entries with K^8^).

**Figure 5:**
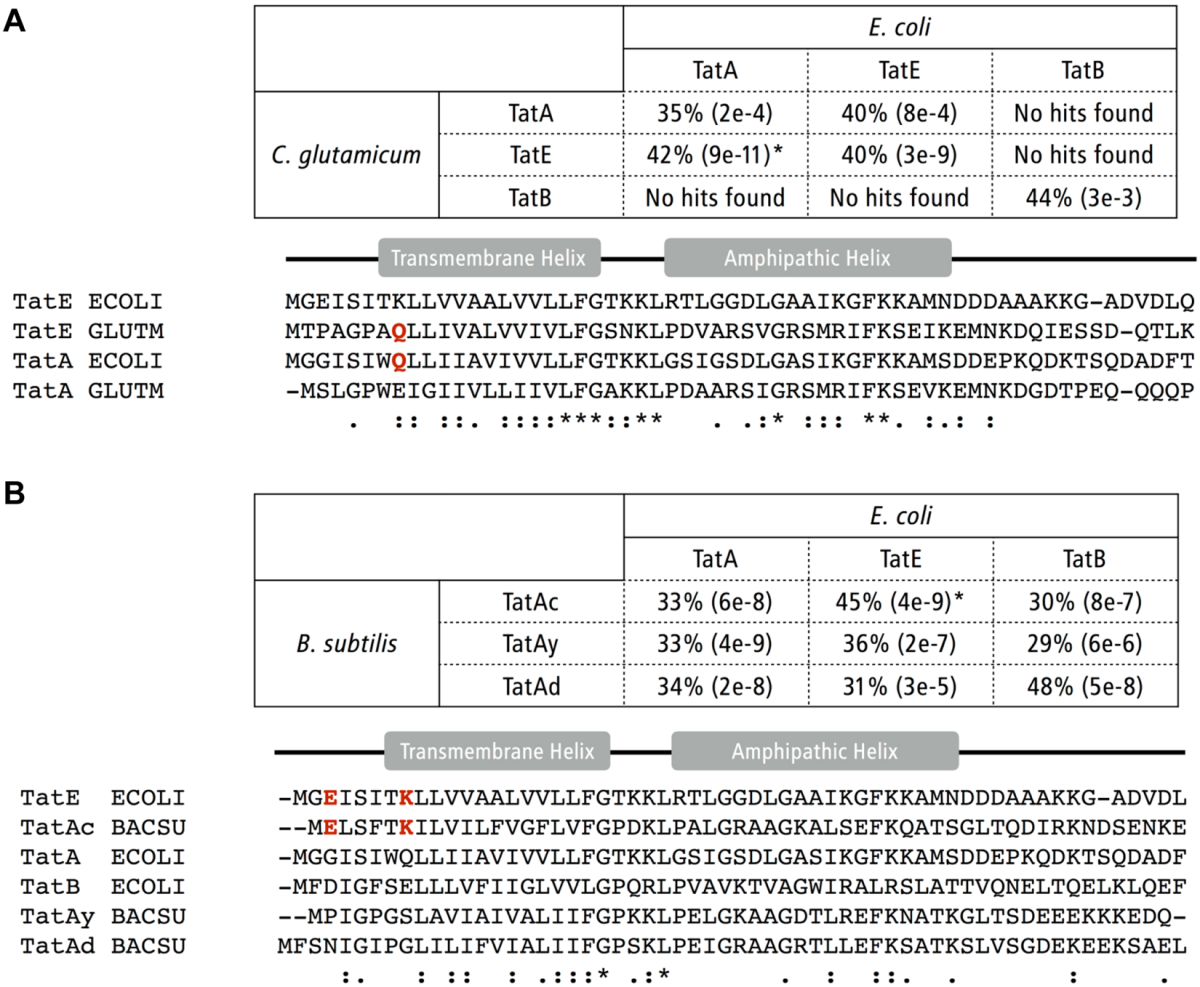
TatE paralogs in Gram-positive bacteria. The degree of sequence similarity, indicated by the percent of identity and the expect e-value (which decreases with the lower chance of random match) between Tat proteins from *Escherichia coli* and *Corynebacterium glutamicum* (**A**) and *Bacillus subtilis* (**B**), respectively, were calculated using NCBI BLAST (upper panels). (**A**) The closest homologue of *C. glutamicum* TatE is TatA from *E. coli*, both featuring non-charged N-termini as revealed in the multiple sequence alignment (lower panel). (**B**) In *B. subtilis*, TatAc is most similar to TatE of *E coli*, as shown by the common ExxxxK motif. Sequence source: *C. glutamicum* TatA (Q8NQE4), TatE (Q2MGW8), TatB (Q8NRD0); *B. subtilis* TatAy (O05522), TatAc (O31804), TatAd (O31467).

A TatE paralog had also been described for the Gram-positive organism *Corynebacterium glutamicum*^34^. In contrast, the inclusion threshold for TatE homologues that we defined for our database search did not yield any result, neither from *C. glutamicum* nor from other Gram-positive organisms. We therefore performed a sequence alignment of the TatABE proteins form *C. glutamicum* and *E. coli* and found that the annotated TatE of *C. glutamicum* actually shares a higher sequence similarity with TatA than with TatE from *E. coli* (Fig. 5A). Moreover, the annotated TatE of *C. glutamicum* lacks any charged amino acids within its N-terminal region but shares the Gln^8^ with *E. coli* TatA (Fig. 5A). However, without further functional analyses, a clear assignment of the two TatA paralogs of *C. glutamicum* to the TatA and TatE families is difficult to undertake.

On the other hand, we found the ExxxxK motif characteristic for *E. coli* TatE also contained in TatAc, which is one of the three annotated TatA paralogs of the Gram-positive bacterium *Bacillus subtilis* (Fig. 5B). Intriguingly, TatAc was recently shown to functionally compensate for mutation-borne defects of TatAy, which is another TatA paralog of *B. subtilis*^35^. TatAc was, however, not able to complement the complete absence of TatAy suggesting that TatAc and TatAy might functionally interact^35^. Thus the minimal Tat translocases of *B. subtilis* consisting of single TatC and TatA components^36,37^ might in fact associate with an auxiliary TatA component. In Fig. 5B we aligned the three TatA paralogs TatAc, TatAy, TatAd of *B. subtilis* with the protein sequences of *E. coli* TatA, TatE, and TatB. Strikingly, the sequence alignment of *B. subtilis* TatAc and *E. coli* TatE, both exhibiting the ExxxxK motif, gave 45% identical amino acid residues within the aligned parts indicating that TatAc might more likely be a functional paralog of *E. coli* TatE than of *E. coli* TatA. Hence, all these sequence compilations demonstrate a wide distribution of TatA-family members that possess a Lys at a position equivalent to the 8^th^ position of enterobacaterial TatE (Fig. 3B). This suggests that unique TatE-type paralogs of TatA might operate in a much larger number of bacterial Tat translocases than previously appreciated.

### TatE-specific interactions with the Tat proteins

Following an analysis of co-evolutionarily predicted residue contacts between TatC and TatA-family proteins as well as the results of molecular dynamics simulations, it was recently proposed that Glu^8^, which is conserved among almost all TatB orthologs, is part of a polar cluster that ligates TatB to TatC in a functional manner^15^. Similarly, Gln or His that populate residue 8 in the vast majority of TatA paralogs would allow TatA to interact with TatC via the same polar cluster^15^. In contrast, the Lys^8^ residue, which obviously is a hallmark of TatE-type orthologs, is likely to mediate different contacts with TatC. This is strongly suggested by the phenotype of an *E. coli* TatB variant, which due to a Glu^8^ to Lys^8^ substitution associates with TatC in a manner that allows proficient recognition of otherwise transport-defective signal peptides^38–40^. By inference, TatA-family members naturally displaying a positively charged residue at position 8, such as most TatE paralogs, are also likely to interact with TatC in a manner different from the mostly negatively charged TatB orthologs. Association of TatE paralogs with TatC could even represent an advanced step in the assembly of a functional Tat translocase following the initial TatBC interaction.

**Figure 6:**
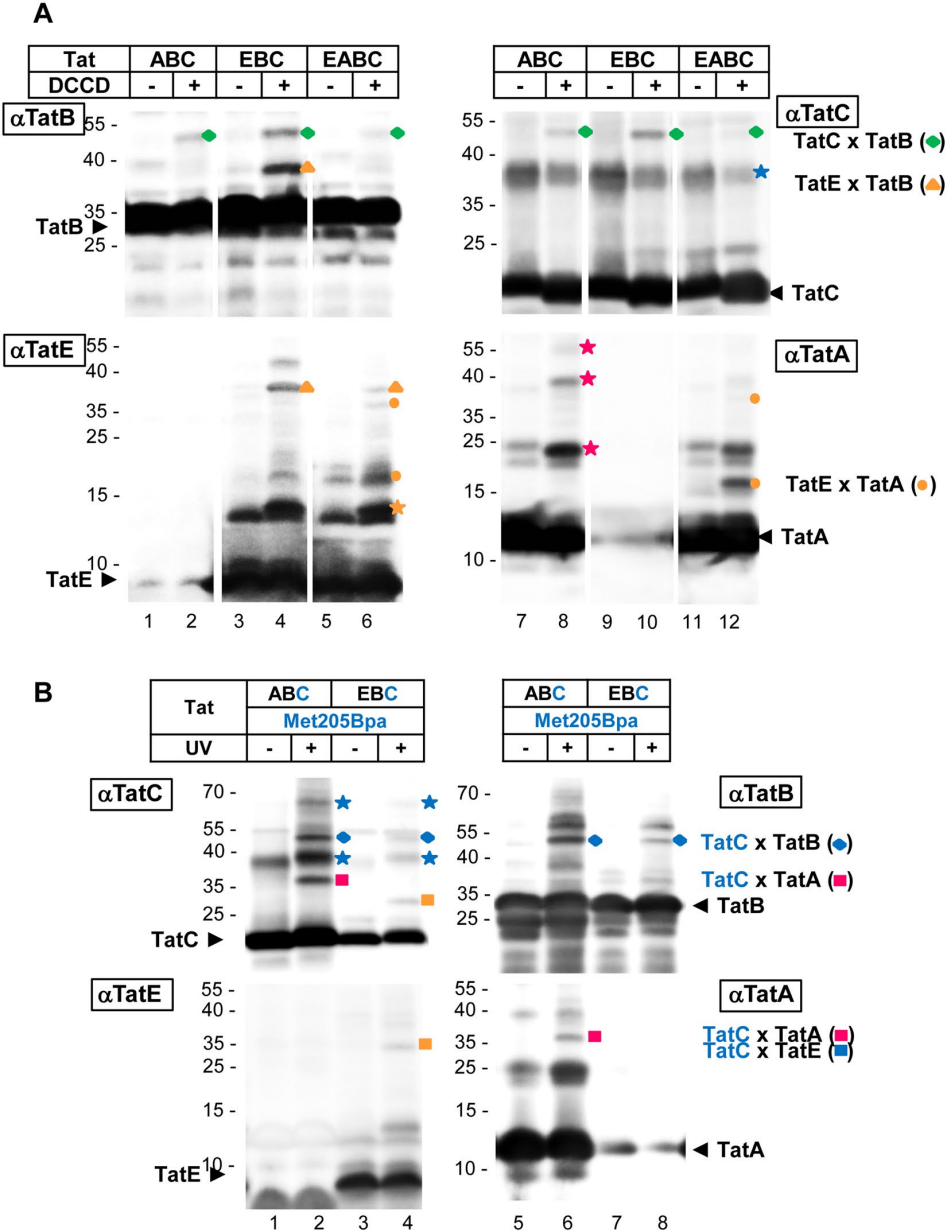
TatE-specific interactions with TatA, TatB and TatC. Western blot analysis of crosslinking experiments with inverted inner membrane vesicles containing the indicated Tat subunits (TatABC, EBC, EABC). After crosslinking, the samples were resolved using 9% Tricine-SDS-PAGE and detected by immunoblotting using antibodies against TatB (αTatB), TatC (αTatC), TatE (αTatE) and TatA (αTatA). (**A**) Crosslinking using *N*,*N*′-dicyclohexylcarbodiimide (DCCD). After incubation with DCCD higher molecular bands representing TatB-TatC adducts (green diamond), TatE-TatB adducts (orange triangle), and TatE-TatA adducts (orange dots) were detected. Indicated are the positions corresponding in size to TatC dimers (blue star), TatE dimers (orange star) and TatA oligomers (magenta stars). Lanes are derived from four individual gels. The gaps mark the excision of single unrelated lanes. (**B**) Crosslinking using *p*-benzoyl-L-phenylalanine (Bpa). During vesicle preparation the crosslinker was incorporated into TatC at position 205 (Met205Bpa). Bpa-crosslinking was induced by exposing the vesicles to UV irradiation. UV-dependent higher molecular bands were identified via their immuno-reactivities as TatC oligomers (blue star), TatB-TatC adducts (blue diamond), TatC-TatA adducts (magenta square) and TatC-TatE adducts (orange square).

In order to obtain more information on how TatE might associate with the other Tat proteins, we explored *N,N*’-dicyclohexylcarbodiimide (DCCD) as a cross-linking agent. DCCD is known to form amide bonds between carboxyl groups located in hydrophobic environments and primary amines^41^. We recently realized that TatB and TatC present in inner membrane vesicles of *E. coli* can be cross-linked in this way (unpublished results). As shown in Fig. 6A, dependent on the addition of DCCD to membrane vesicles containing TatABC a ~45 kDa product appears (green diamond), which is recognized by anti-TatB and anti-TatC antibodies (αTatB, lane 2; αTatC, lane 8). If the vesicles contain TatE instead of TatA (TatEBC), DCCD treatment results in an additional cross-link, ~37 kDa in size, which is recognized by anti-TatB and anti-TatE antibodies (lane 4, orange triangle, αTatB, αTatE). We therefore conclude that in these vesicles, TatE is so closely spaced to TatB that both proteins can be cross-linked by DCCD. This is consistent with previously identified contact sites between TatE and TatB, which photo cross-linking revealed in the transmembrane and amphipathic helices of TatE^28^. An immediate proximity between TatE and TatB within the substrate receptor complex is further reflected by the findings of Fig. 1 demonstrating that in the absence of the H^+^-motive force, the same sites of the TorA signal peptide contact both TatB and TatE.

We next asked where on TatC such a TatB-TatE heterodimer could be located. As to TatB, several recent studies have identified the transmembrane helix 5 of TatC as a docking site for the transmembrane helix of TatB^5,14,15,42^. One of the residues located in transmembrane helix 5 of TatC that repeatedly was shown to mediate contacts with TatB is methionine 205. We therefore incorporated Bpa into *E. coli* TatC at position 205 using an *in vivo* amber stop codon approach and prepared membrane vesicles that carried the TatC^205B*pa*^ mutant in the presence of TatAB and TatEB (Fig. 6B). In the context of the TatAB proteins, TatC^205B*pa*^ when activated by UV light yielded four prominent adducts (lane 2, αTatC). Adducts running at ~65 and ~40 kDa (blue stars) were recognized only by antibodies directed against TatC and therefore represent dimers and trimers of TatC^14,42^. The ~50 kDa cross-linking product (blue diamond) was also recognized by anti-TatB antibodies (αTatB, lane 6) confirming this known contact site of TatC for TatB. The ~37 kDa adduct of TatC^205B*pa*^ (red square) was also detected by anti-TatA antibodies (αTatA, lane 6) consistent with the previously established overlap of the TatA and TatB cross-linking sites on transmembrane helix 5 of TatC^14,15,43^. In vesicles containing TatC^205B*pa*^ together with TatEB instead of TatAB, the UV-dependent cross-links were weaker owing to a lower TatC content of those vesicles (αTatC, compare lanes 2 and 4). As expected, the TatA adduct (red square) was no longer visible. Instead a new ~30 kDa cross-link of TatC^205B*pa*^ to TatE appeared (orange square in αTatC, αTatE, lane 4), indicating that TatE, exactly as TatB and TatA, contacts TatC via its fifth transmembrane helix. Previously it was found that Bpa when incorporated into TatE at position 9 of its transmembrane helix cross-links to TatC^28^. It is therefore reasonable to assume that the contact of TatE with residue 205 in helix five of TatC reflects the alignment of both transmembrane helices as demonstrated before for TatB and TatA^5,24,42^. However, as discussed above the oppositely charged transmembrane helices of TatE and TatB are not likely to occupy exactly the same binding sites on TatC.

Interestingly, the DCCD-mediated contact between TatB and TatE (Fig. 6A, lane 4, orange triangle), was almost gone in the presence of TatA (lane 6). Instead membrane vesicles containing all four Tat components gave rise to new adducts that by size and immuno-reactivity represent TatE-TatA oligomers (orange dots, compare lanes 4 and 6, 10 and 12). If this reflects a true exchange of TatB for TatA as the binding partner of TatE following the recruitment of TatA to the substrate-bound TatBC receptor complex, is speculative at this point of time. The DCCD-mediated cross-links that we obtained between TatE and TatA, however, suggest that TatE could fulfil a role in the recruitment of TatA to the TatBC receptor complex through hetero-oligomerization with TatA. Because *in vivo*, TatE is present only in sub-stoichiometric amounts compared with TatA^20^, TatE could conceivably function as a nucleation point for a TatBC-dependent oligomerization event of TatA.

In conclusion, our results suggest that TatE paralogs have overlapping functions between TatA and TatB. Similar to TatB, TatE is involved in substrate binding yet possibly at a later step. Although it docks at transmembrane helix five of TatC much like TatA and TatB do, the individual interacting epitopes of TatC are likely to vary. We propose that TatE could play a role in the oligomerization of TatA.

## Methods

### Plasmids

The plasmids used in this study are listed in Table S1. To construct plasmid pEC, vector pEBC_LinkRBS was used as a template. Primers flanking *tatB* were designed (pECKI for and rev, Table S1) and phosphorylated. The *tatB* gene was deleted during vector amplification. The PCR product was purified and ligated prior to transformation. Plasmid pEBC_LinkRBS was also used to prepare the amber stop codon mutant at position Met205 in *tatC* in the context of *tatEB* using mutagenesis PCR^19^ and the primers Met205 for and rev listed in Table S1. Construction of the same TatC variant in the context of *tatAB* was previously described^14^.

### *In vitro* reactions

The plasmid-encoded Tat substrate TorA-MalE335 and its Bpa variants were synthesized in a cell free transcription/translation system in 50 μl reactions^44^. Cell extracts were prepared using *E. coli* strain SL119^45^ or Top10 (Invitrogen) transformed with plasmid pSup-BpaRS-6TRN(D286R) for Bpa incorporation into TorA-MalE335. The vesicles were added 12 min after starting protein synthesis at 37 °C and incubated for 18 min at 37 °C. In order to disrupt the proton motive force, 0.1 mM CCCP was added at the onset of synthesis. After incubation with vesicles, Bpa crosslinking was induced by UV irradiation for 20 min on ice. To visualize transport of TorA-MalE335 into the vesicles, completed reactions were treated with 0.5 mg/ml Proteinase K.

For immuno-precipitation, the samples were denatured in 1% SDS for 10 min at 95 °C after crosslinking. Antisera against TatA, TatE and TatB were incubated with Protein A-Sepharose beads for 90 min at 4 °C. Denatured samples were cleared by brief centrifugation and applied to the antibody-loaded Protein A-Sepharose during 3 h at 4 °C on a spinning wheel. After 4 washing steps, antibody-bound material was released by incubating in SDS-PAGE loading buffer for 10 min at 37 °C and 1.400 rpm.

The proteins were resolved on SDS-PAGE using 10% polyacrylamide gels. Radioactive gels were developed by phosphorimaging using a Storm 845 instrument. Quantification was performed using ImageQuantTL.

### Inverted inner membrane vesicles

Vesicles were prepared according to^44^ from *E. coli* strains DADE (MC4100*∆tatABCDE*)^46^ or Bl21(DE3)∆Tat (B. Ize and T. Palmer, personal communication) expressing the vectors pBADxTat (TatABC), pEBC_LinkRBS (TatEBC), pEABC_LinkRBS (TatEABC), p8737-tatAC (TatAC), pFAT75CH∆A (TatBC) or pECKI (TatEC), respectively. Vesicles containing TatABC^Met205Bpa^ were prepared from BL21(DE3) *E. coli* cells transformed with pEVOL-pBpF and p8737, and vesicles containing TatEBC^Met205Bpa^ from DADE cells containing pEVOL-pBpF and pEBC_LinkRBS. Expression of plasmid-encoded genes was induced by 0.1% arabinose or 1 mM IPTG (p8737-tatAC and pFAT75CH∆A). The concentration of the vesicles was adjusted to OD_280_ ≈ 100.

To detect crosslinks between the Tat subunits, 5 µl vesicles were diluted with 95 µl INV-buffer^44^. When indicated 1 µl DCCD (50 mM) was added. The samples were exposed to UV light for 20 min on ice, precipitated with trichloroacetic acid and resuspended in 100 µl Tricine sample buffer^47^. Proteins were then resolved using 9% Tricine SDS-PAGE and identified by Western blotting using antibodies against TatE (10 µl sample per lane), TatA (7 µl per lane), TatB and TatC (20 µl per lane).

### Protein sequence analysis

All sequences used in this research were obtained from Uniprot or NCBI Reference Sequence (RefSeq) protein databases. The NCBI RefSeq protein database^48^ release 79 was queried with the TatE from *Escherichia coli* using BLASTP^49^ with cut-off e-value set to 1e-3. After removing incomplete entries, 3120 sequences were obtained. Among them, the 111 enterobacterial homologues were aligned with Clustal Omega^50^ and the consensus sequence logo was plotted using WebLogo3^51^. The degree of sequence similarity of Tat proteins between *E. coli* (TatA, TatE, TatB) and *C. glutamicum* (TatA, TatE, TatB) or *B. subtilis* (TatAy, TatAc, TatAd) were investigated by pairwise-alignment using NCBI BLAST.

### Data availability

All data generated or analysed during this study are included in this published article (and its Supplementary Information files).

## Electronic supplementary material

Supplementary Dataset 1
