# Supplementary material for: Unanticipated functional diversity among the TatA-type components of the Tat protein translocase
Source: Sci Rep. 2018 Jan 22;8:1326. doi: 10.1038/s41598-018-19640-3 (PMC5777986; doi:10.1038/s41598-018-19640-3)
Supplement: Supplementary file 1 — Supplementary Dataset 1 [file 41598_2018_19640_MOESM1_ESM.doc]

**Supplementary Information**

**Unanticipated functional diversity among the TatA-type components of the Tat protein translocase**

**Ekaterina Eimer, Wei-Chun Kao, Julia Fröbel, Anne-Sophie Blümmel, Carola Hunte and Matthias Müller**

**Supplementary Table S1.** **Plasmids and primers used.**

| **Plasmid** | **Vector** | **Insert** | **Reference** |
| --- | --- | --- | --- |
| pPJ1 | pET22b+ | TorA-MalE335 |  |
| pPJ1_F14Bpa | pET22b+ | amber stop codon in TorA-MalE335 |  |
| pPJ1_V23Bpa | pET22b+ |
| pPJ1_L27Bpa | pET22b+ |
| pPJ1_P34Bpa | pET22b+ |
| p8737 | pET22b+ | TatABC |  |
| p8737-tatAC | pET22b+ | TatAC |  |
| pFAT75CH∆A | pQE60 | TatBC^His^ |  |
| pEC | pBAD33K | TatEC^His^ | This study |
| pEBC_LinkRBS | pBAD33K | TatEBC |  |
| pEABC_LinkRBS | pBAD33K | TatEABC |
| pBADxTat | pBAD33K | TatABC |
| pSup-BpaRS-6TRN(D286R) |  |  |  |
| pEVOL-pBpF |  |  |  |

| **Primer** | **Sequence** |
| --- | --- |
| TatC205 for | CATTCGTTGTCGGG**TAG**TTGCTGACGCCGC |
| TatC205 rev | GCGGCGTCAGCAA**CTA**CCCGACAACGAATG |
| pECKI for | ACATGTATGTCTGTAGAAGATACTCAACCGCTTATC |
| pECKI rev | TCCTCTCGTGAGCTCGAATTCGTTAGC |

**Supplementary References**

1. Zoufaly, S., Fröbel, J., Rose, P., Flecken, T., Maurer, C., Moser, M., and Müller, M. (2012) Mapping Precursor-binding Site on TatC Subunit of Twin Arginine-specific Protein Translocase by Site-specific Photo Cross-linking. *J Biol Chem* **287**, 13430-13441

2. Blümmel, A. S., Haag, L. A., Eimer, E., Müller, M., and Fröbel, J. (2015) Initial assembly steps of a translocase for folded proteins. *Nat Commun* **6**, 7234

3. Alami, M., Trescher, D., Wu, L. F., and Müller, M. (2002) Separate analysis of twin-arginine translocation (Tat)-specific membrane binding and translocation in Escherichia coli. *J Biol Chem* **277**, 20499-20503

4. Fröbel, J., Rose, P., Lausberg, F., Blümmel, A. S., Freudl, R., and Müller, M. (2012) Transmembrane insertion of twin-arginine signal peptides is driven by TatC and regulated by TatB. *Nat Commun* **3**, 1311

5. Orriss, G. L., Tarry, M. J., Ize, B., Sargent, F., Lea, S. M., Palmer, T., and Berks, B. C. (2007) TatBC, TatB, and TatC form structurally autonomous units within the twin arginine protein transport system of Escherichia coli. *FEBS Lett* **581**, 4091-4097

6. Eimer, E., Fröbel, J., Blümmel, A. S., and Müller, M. (2015) TatE as a Regular Constituent of Bacterial Twin-arginine Protein Translocases. *J Biol Chem* **290**, 29281-29289

7. Ryu, Y., and Schultz, P. G. (2006) Efficient incorporation of unnatural amino acids into proteins in *Escherichia coli*. *Nat Methods* **3**, 263-265

8. Young, T. S., Ahmad, I., Yin, J. A., and Schultz, P. G. (2010) An enhanced system for unnatural amino acid mutagenesis in *E. coli*. *J Mol Biol* **395**, 361-374
